# Qishen Yiqi alleviates periostin-mediated cardiac fibrosis and hypertrophy in Dahl hypertensive rat hearts and angiotensin II-induced cardiac organoids

**DOI:** 10.3389/fphar.2025.1491582

**Published:** 2025-06-18

**Authors:** Siwen Fan, Hongxia Du, Siyu Li, Guangxu Xiao, Yuhan Zhao, Shuang He, Guanwei Fan, Yan Zhu

**Affiliations:** ^1^ State Key Laboratory of Component-Based Chinese Medicine, Tianjin University of Traditional Chinese Medicine, Tianjin, China; ^2^ Haihe Laboratory of Modern Chinese Medicine, Tianjin, China; ^3^ Tianjin Key Laboratory of Translational Research of TCM Prescription and Syndrome, First Teaching Hospital of Tianjin University of Traditional Chinese Medicine, Tianjin, China

**Keywords:** QSYQ, salt sensitive hypertension, cardiac spheroid, heart damage, periostin

## Abstract

**Background:**

Arterial hypertension is a significant risk factor for cardiovascular health. Long-lasting hypertension leads to damage to multiple organs, such as the heart, kidneys, and vascular bed damage. We have previously shown that a component-based Chinese medicine Qishen Yiqi (QSYQ) lowered the blood pressure and ameliorated kidney damage in salt-sensitive hypertensive rats. However, its effect on the hypertensive rat heart remains unknown. This study aims to explore the efficacy and mechanism of QSYQ in hypertensive heart disease.

**Methods:**

Dahl salt-sensitive hypertension rats were fed with normal or high-salt diets with gavage administration of QSYQ or control drug for 9 weeks. Cardiac ultrasound, tissue pathology and transcriptome analysis were performed on the hypertensive heart *in vivo*. A cardiac spheroid model we established previously was treated with angiotensin II to mimic a hypertensive heart *in vitro*.

**Results:**

QSYQ prevented the development of diastolic dysfunction of LVPW and E/A and reduced fibrosis and hypertrophy in the hypertensive rat hearts. In cardiac spheroids, angiotensin II induced an exacerbated hypertrophic morphology, fibrotic pathology, and elevated collagen expression. QSYQ treatment effectively reversed these abnormalities. Transcriptome analysis revealed that periostin is a key target of QSYQ in the hypertensive heart. Consistently, QSYQ also significantly downregulated the expression of periostin and fibrosis indicators such as TGF-β, *α*-SMA, Col1a1 and Col3a1.

**Conclusion:**

QSYQ alleviates cardiac fibrosis and hypertrophy in Dahl Salt-sensitive hypertension rats *in vivo* and angiotensin II-induced cardiac organoids *in vitro* via regulating multiple signaling pathway activator periostin.

## Graphical Abstract

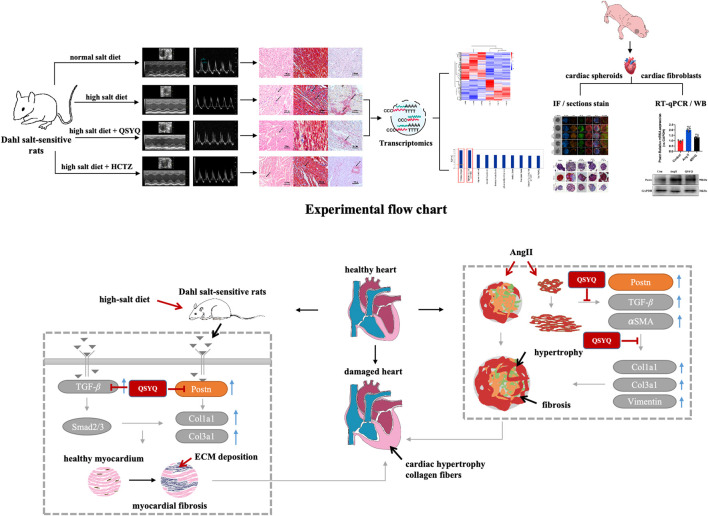

## 1 Introduction

Hypertensive and heart disease complications fuel cardiovascular morbidity and mortality. Cardiovascular diseases, especially hypertension, have been established to change the tissue structure and composition of the left ventricle (LV) and ultimately change the mechanical behavior (Grobbel et al., 2021). Long-term exposure to high pressure can lead to pathological features such as cardiomyocyte hypertrophy, fibrosis, and increased resting tension of myocytes in the left ventricle, leading to centripetal hypertrophy (Borlaug, 2020; Mishra and Kass, 2021; Nair, 2020). Although antihypertensive drugs can control blood pressure, it is difficult to protect target organs from long-term damage completely. Therefore, the ability to effectively reduce heart damage while maintaining blood pressure remains to be resolved.

A long-term high salt diet is one of the most critical environmental factors for hypertension (Bier et al., 2018; Rucker et al., 2018). The evidence has revealed that excessive dietary salt in rats can lead to coronary artery perivascular fibrosis, ventricular interstitial fibrosis, ventricular ischemia and ventricular diastolic dysfunction (Gao et al., 2011). A key aspect of hypertensive heart disease is cardiac fibrosis. Fibrosis, a surplus extracellular matrix, is detrimental that promotes contractile dysfunction. However, due to the complex pathological process of hypertensive heart disease, cardiovascular medicines with a single mechanism are unable to provide systematic and thorough heart protection. Therefore, these medical challenges must be solved if more patients are to stay away from the life-threatening risks caused by hypertensive heart disease and improve their quality of life. Traditional Chinese medicine has few side effects on cardiovascular disease and contains the synergistic effect of a variety of active and effective compounds, so TCM has shown great advantages in the treatment of heart damage with hypertension.

Qishen Yiqi (QSYQ) is a China State Food and Drug Administration-approved component-based Chinese medicine. It consists of Astragalus, Salvia miltiorrhiza, Panax notoginseng, and Dalbergia odorifera, blended in accordance with TCM theory of invigorating the qi, enhancing blood circulation and dissipating blood stasis. Clinically, it is mainly used to treat coronary heart disease, angina pectoris and secondary prevention of myocardial infarction. It has been reported that the main components of QSYQ include danshensu, rosmarinic acid, protocatechualdehyde, calycosin-7-O-β-D-glucoside, and ononin (Xie et al., 2021). Studies have shown that QSYQ improves ischemia/reperfusion-induced myocardial fibrosis by regulating TGFβ1/Smads signaling pathway (Zheng et al., 2019). QSYQ and its components have a protective effect on myocardial ischemia by regulating energy metabolism (Cui et al., 2018). QSYQ alleviates cardiac hypertrophy and dysfunction caused by fatigue by modulating energy metabolism (Huang et al., 2019). Our previous studies showed that QSYQ could reduce blood pressure and improve kidney damage in Dahl salt-sensitive rats (Du et al., 2021). However, the cardioprotective effect of QSYQ on Dahl salt-sensitive rats has not been explored. Therefore, this article mainly studies QSYQ on the protective potential of heart injury in salt-sensitive hypertension rats.

Uncontrolled blood pressure leads to myocardial remodeling. Fibrosis is the pathophysiological basis of hypertension and ultimately leads to heart failure. Myocardial fibrosis is considered an early event of heart failure. Fibrosis is caused by fibrotic factors, collagen synthesis and degradation, upregulation of extracellular matrix and increased oxidative stress. Periostin (POSTN) is a 90 kDa peptide secreted from the resident cardiac fibroblasts and myofibroblasts located in the fibrous barrier between the valve, atrium, ventricle, and ventricular myocardium. The main structure of the peptide is four repeated fasciclin (Litvin et al., 2006), which can promote the process of fibrosis by regulating ECM homeostasis (Liu et al., 2014). POSTN has very low expression levels in heart-healthy mesenchymal cells, including valve interstitial cells, vascular smooth muscle cells and cardiac fibroblasts. However, POSTN is essential in activating fibroblasts to secrete proteome in damaged hearts (Kanisicak et al., 2016). Studies have shown that the upregulation of POSTN expression in human failing hearts is related to myocardial fibrosis (Zhao et al., 2014). Another study conducted on mice after transected aorta narrowing confirmed the role of POSTN in heart failure (Liu et al., 2013). However, the therapeutic implications of POSTN modulation remain paradoxical in hypertensive cardiomyopathy pathogenesis. Notably, while these findings establish POSTN as a biomarker of advanced fibrotic deterioration, current clinical evidence remains insufficient to validate its direct cardioprotective potential against hypertension-induced myocardial injury. All in all, POSTN may play a vital role in the prevention of hypertensive heart damage.

Critical to understanding the biology of hypertension and associated cardiovascular disorders is the generation of angiotensin II (Ang II) (Zoungas and Asmar, 2007). Ang II increases arterial pressure by inducing intense vasoconstriction and promoting the reuptake of sodium and water in the renal tubules, increasing arterial pressure and damaging the heart. Based on previous research, experimental models of Ang II-induced hypertension are accompanied by the occurrence of cardiac remodeling processes (Johar et al., 2006). Furthermore, Ang II is the most important hormone for regulating myocardial fibrosis (Bai et al., 2013; Kuwabara et al., 2022), and previous studies have shown that Ang II antagonists can prevent fibrosis in various diseases (Klein et al., 2017; Shenoy et al., 2010). Therefore, it is reasonable to use a vitro model induced by Ang II to explore the study of QSYQ on hypertensive cardiac fibrosis. However, the structure of the heart is complex, consisting of multiple layers of tissue and different types of cells that work together to ensure the proper functioning of the heart (Hofbauer et al., 2021). However, the two-dimensional (2D) cell, the most widely and traditionally used *in vitro* cell models of the heart, is difficult to reproduce *in vivo* environments and supply the three-dimensional (3D) models needed for cell-cell and cell-matrix interactions (Lee et al., 2020). Combining both 2D and 3D cardiac spheroid *in vitro* models suppotrs not only 3D cell-cell and cell-matrix interactions but also performs multi-dimensional model evaluation, so that it is an ideal model to study cardiac fibrosis *in vitro*.

In this study, utilizing Dahl salt-sensitive rat models with 3D cardiac spheroids and 2D fibroblasts, we demonstrated that QSYQ attenuates hypertensive cardiac remodeling by suppressing POSTN-driven fibrotic signaling. Mechanistically, QSYQ treatment: (1) downregulated pathological POSTN overexpression in pressure-overloaded myocardium, (2) inhibited fibroblast activation in spheroids, and (3) reduced collagen hypersecretion *in vitro*. These multi-dimensional analyses establish QSYQ’s cardioprotection as being mediated through POSTN inhibition, repositioning this traditional formulation as a targeted modulator of fibrotic pathways. Our findings provide mechanistic justification for QSYQ’s therapeutic application in hypertensive cardiomyopathy via strategic interception of POSTN-mediated maladaptive remodeling.

## 2 Materials and methods

### 2.1 Drugs and reagents

Qishen Yiqi was provided by Tasly Pharmaceutical Group Co., Ltd. For a detailed description, please refer to the Supplementary Material for the Materials and Methods section.

### 2.2 Animals

Seven-week-old male Dahl salt-sensitive rats (Dahl SS) were purchased from Beijing Vital River Lab Animal Technology Co., Ltd. (Beijing, China, Certificate No.: SCXK Jing 2016-0006) and allowed to drink and eat freely. The animal research adhered to guidelines from China’s Ministry of Science and Technology and received approval from the Laboratory Animal Ethics Committee at Tianjin University of Traditional Chinese Medicine (License No: TCM-LAEC2014004). Details on animal grouping and drug administration are provided in the Data Supplement.

### 2.3 Isolation and preparation of cardiac fibroblasts and 3D cardiac spheroid

Cardiac fibroblasts (CFs) were isolated from 1-3-day-old neonatal Sprague Dawley (SD) rats by quickly cutting the neonatal hearts without atria quickly in ice-cold PBS. Minced cardiac tissue was sequentially incubated for 5 min each at 37°C with 0.25% trypsin and 0.1% collagenase solutions. Cardiac cell suspensions were collected in M199 medium on ice, filtered and centrifuged. The cardiac fibroblasts were plated after 1.5–2 h and the medium was changed to Dulbecco’s modified Eagle medium (DMEM). 3D cardiac spheroids were obtained as previously described (Fan et al., 2023). Briefly, cardiac cell including CFs, cardiomyocytes (CMs), and endothelial cells (ECs) were co-cultured in M199 medium at 1 × 10^5^ cells/well in 96-well round-bottom plates (MS-9096UZ, Sumitomo Bakelite Co., Ltd., Tokyo, Japan), with medium changes every 2 days. Experiments proceeded once 3D cardiac spheroids formed.

### 2.4 Echocardiography, transcriptome sequencing, core analysis of differentially expressed genes (DEGs), Real-time reverse transcription polymerase chain reaction (RT-PCR) assay, ELISA, immunofluorescence (IF), histology, western blotting (WB)

Complete primer sequences for the quantitative polymerase chain reaction are included in Supplementary Table S1 of the Data Supplement. The Supplement contains the methods and software used in Echocardiography, Transcriptome sequencing, Core Analysis of Differentially Expressed Genes (DEGs) and the antibodies and techniques used in Western blotting, ELISA, Histology, and immunohistochemistry.

### 2.5 Statistical analysis

All data were expressed as mean ± SEM. Statistical analysis was performed using GraphPad Prism 7 software (GraphPad Software, Inc., La Jolla, CA, United States). Students’ two-tailed t-test was used to compare the two groups, and the One-way analysis of variance (ANOVA) was used to compare three or more groups. There was a correction for multiple comparisons following ANOVA. A value of P < 0.05 was considered statistically significant.

## 3 Results

### 3.1 Effects of QSYQ on diastolic function in Dahl salt-sensitive rats

**FIGURE 1:**
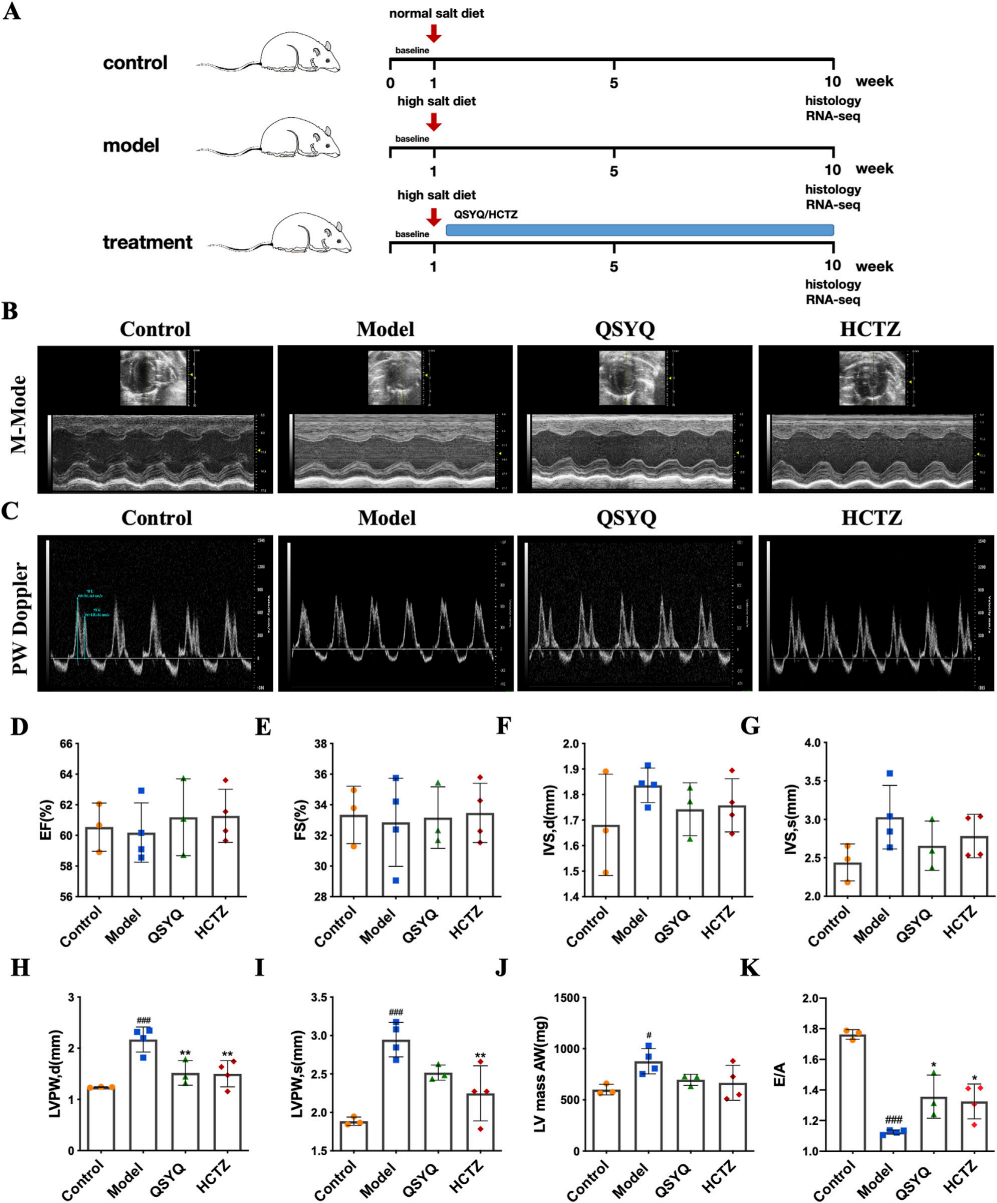
QSYQ prevented the development of diastolic dysfunction in hypertensive Dahl rats. **(A)** The experimental scheme. **(B)** Representative images of left ventricular echocardiography of each group of rats. **(C)** Representative photos of the apical four-chamber view of the heart pulse wave PW Doppler of each group of rats. Quantification of echocardiographic changes in cardiac function in each group: **(D)** EF, **(E)** FS, **(F)** LVSd, **(G)** LVSs, **(H)** LVPWd, **(I)** LVPWs and **(J)** LV mass (*n* = 3–4 in each group). **(K)** Quantification of E/A peaks in each group of rats (*n* = 3–4). Data were expressed as mean ± SEM. ^#^P < 0.05, ^###^P < 0.001 vs. Control; **P < 0.01, **P < 0.01 vs. Model.

Dahl salt-sensitive rats were given a high-salt diet for 5 weeks to induce a model of hypertensive heart disease. After that, the pharmacological intervention was done while high-salt feeding continued (Figure 1A). The effects of QSYQ on cardiac function were first examined by echocardiography (Figures 1B,C). Nine weeks of a high-salt diet decreased EF% and FS% (60.19 ± 1.94 vs. 60.54 ± 1.57 and 32.85 ± 2.88 vs. 33.34 ± 1.87, was a downward trend, but it did not reach statistical significance) and increased LVSd and LVSs in Dahl salt-sensitive rats (1.83 ± 0.07 vs. 1.68 ± 0.2 and 3.03 ± 0.41 vs. 2.44 ± 0.24), and treatment with QSYQ increased EF% and FS% (61.19 ± 2.51 vs. 60.19 ± 1.94 and 33.34 ± 1.87 vs. 32.85 ± 2.88), and decreased LVSd and LVSs (1.74 ± 0.1 vs. 1.83 ± 0.07 and 2.67 ± 0.32 vs. 3.03 ± 0.41). It is important to note that EF and FS only have a trend in treatment, but no significant effect. Compared with the Control, neither QSYQ nor HTCZ treatment significantly affected LVSd and LVSs (Figures 1D–G). Interestingly, LVSd and LVSs in QSYQ-treated rats were better than those of HCTZ (1.74 ± 0.1 vs. 1.76 ± 0.1 and 2.67 ± 0.32 vs. 2.78 ± 0.28) (Figures 1F,G). The cardiac function parameters LVPWd in the QSYQ- and HTCZ-treated rats were significantly improved compared with the Model (2.17 ± 0.25 vs. 1.52 ± 0.24 and 2.17 ± 0.25 vs. 1.50 ± 0.25) (Figure 1H). Furthermore, compared with the Model, QSYQ had no significant effect on LVPWs (2.95 ± 0.22 vs. 2.52 ± 0.1) (Figure 1I). Compared with the Control rats, LV mass was significantly increased (877.82 ± 123.61 vs. 600.41 ± 51.84), and there was a downward trend in the QSYQ and HTCZ treatment groups (696.64 ± 53.8 vs. 877.82 ± 123.61 and 667.25 ± 170.41 vs. 877.82 ± 123.61), but it did not reach statistical significance (Figure 1J). Compared with the Control, the E/A peak of the high-salt fed Dahl rats was significantly lower, indicating a diastolic dysfunction (1.76 ± 0.03 vs. 1.12 ± 0.01) (Figure 1K). However, both QSYQ and HCTZ dramatically improved the diastolic dysfunction (1.36 ± 0.14 vs. 1.12 ± 0.01 and 1.32 ± 0.11 vs. 1.12 ± 0.01) (Figure 1K). These findings indicate that QSYQ improved cardiac function in hypertensive Dahl rats.

### 3.2 Impact of QSYQ on histopathological alterations in Dahl rat heart

**FIGURE 2:**
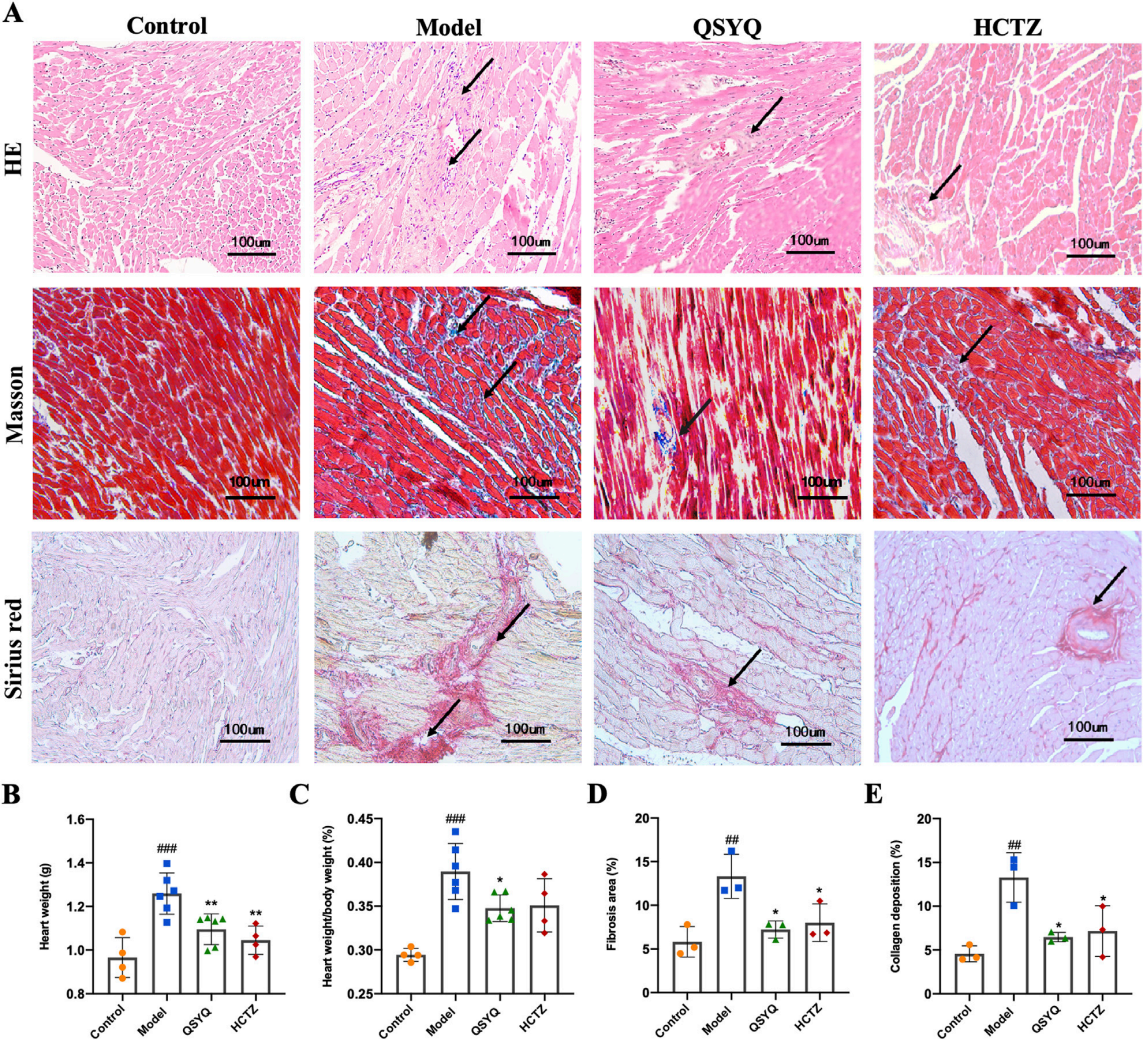
QSYQ reduced cardiac hypertrophy and fibrosis in Dahl hypertensive rat hearts. **(A)** Representative images of H&E, Masson and Sirius red staining of heart tissue in each group. Black arrows indicate the pathological changes, the area of fibrosis and the area of myocardial interstitial fibrosis. **(B)** Heart weight in each group of rats (*n* = 4–6). **(C)** The ratio of heart weight to body weight (*n* = 4–6. **(D)** Quantification of the area of myocardial interstitial fibrosis in each group (*n* = 3). **(E)** Quantification of collagen deposition area in myocardial interstitium in each group (*n* = 3). Data were expressed as mean ± SEM. ^##^P < 0.01, ^##^P < 0.001 vs. Control; *P < 0.05, **P < 0.01 vs. Model.

H&E staining revealed that compared to the Control, the Model group’s heart tissue exhibited cardiomyocyte degeneration, necrosis, and inflammation (Figure 2A). In addition, cardiomyocytes in the Model were hypertrophied, distributed in strips, and the myocardial interstitium widened. In comparison, inflammatory infiltration of the heart tissue in QSYQ- and HCTZ-treated rats was reduced, and the cardiomyocytes were arranged neatly (Figure 2A). QSYQ and HCTZ treatment reduced inflammation and arranged the cardiomyocytes more orderly. Heart weight and hypertrophy were significantly lessened by QSYQ (Figures 2B,C). Masson and Sirius red staining (Figure 2A) showed that QSYQ and HCTZ markedly decreased cardiac fibrosis and collagen in hypertensive rats (Figures 2D,E), suggesting their effectiveness in mitigating pathological changes and hypertrophy due to high salt-induced hypertension.

### 3.3 Transcriptome analysis of differentially expressed genes (DEGs) revealed potential targets of QSYQ in protecting salt-sensitive hypertension-induced heart damage

**FIGURE 3:**
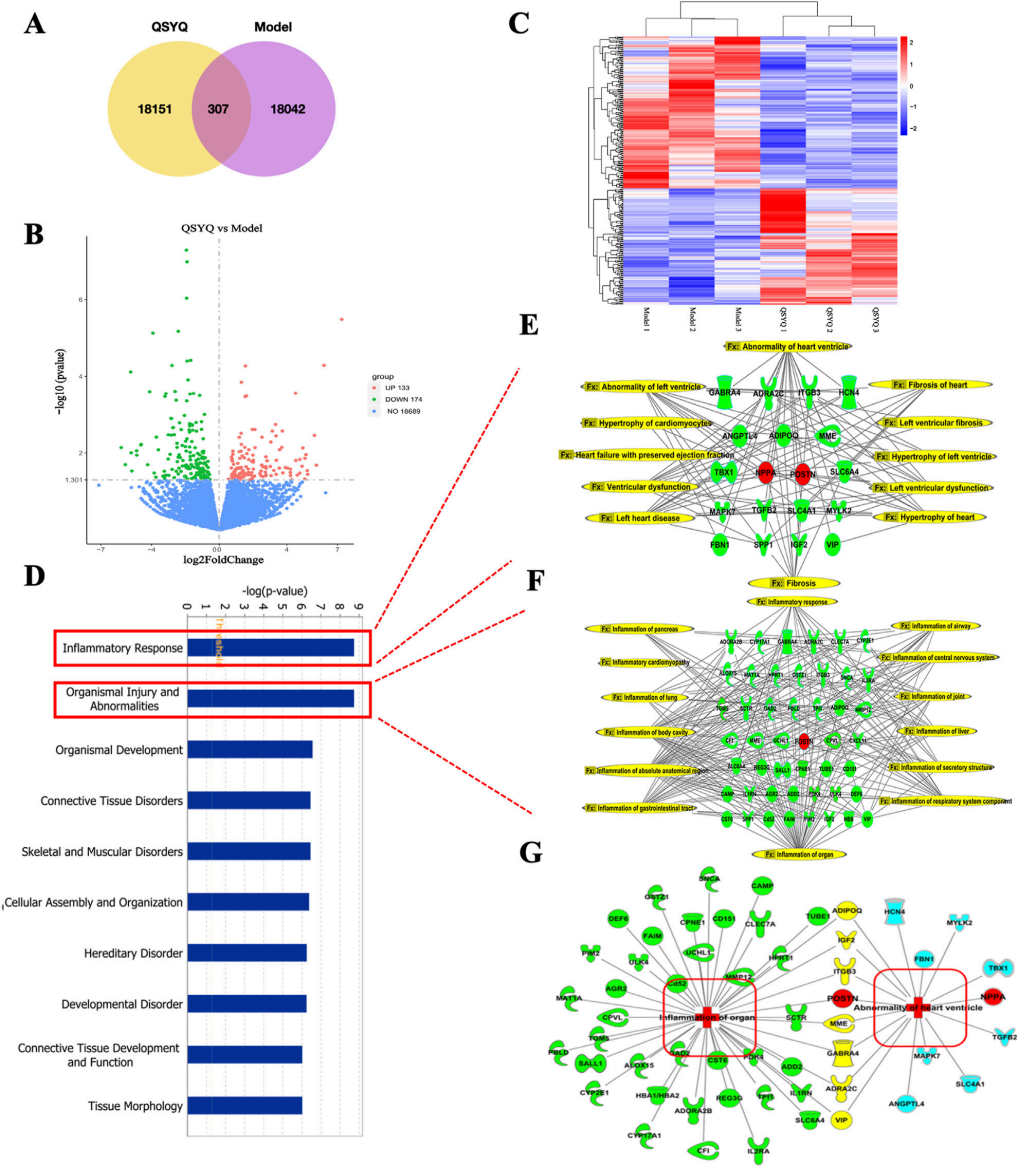
Transcriptome sequencing and IPA analyses revealed major pathways affected by QSYQ in Dahl hypertensive rat hearts. **(A)** Venn diagram of QSYQ group vs. Model group. **(B)** Volcano map of differentially expressed genes regulated by QSYQ treatment. The differentially expressed genes in the test sample were presented with different colors. Red represents genes with upregulated expression, green represents genes with downregulated expression, and blue represents genes with no difference in expression. **(C)** Hierarchical cluster analysis between samples from the QSYQ group and Model groups (*n* = 3). X-axis was the sample duplicates, and Y-axis listed the differentially expressed genes. **(D)** The diseases and functions affected by QSYQ were ranked according to Fisher’s exact test algorithm. The top 10 functions were sorted in descending order of -log (p-value) score. Inflammation response and Organismal injury and abnormalities ranked first and second among them (in red boxes). **(E)** The 19 QSYQ targets correlated with an abnormality of the heart ventricle from organismal injury and abnormalities. **(F)** The 47 QSYQ targets correlated with inflammation of organs from inflammation response. **(G)** The relationship between inflammation of organ-related targets (green) and abnormality of heart ventricle-related targets (blue). The common targets shared by the inflammation of the organ and abnormality of the left ventricle were displayed in yellow.

RNA sequencing was performed on heart tissues from Dahl salt-sensitive rats on a high-salt diet, with and without QSYQ treatment, to identify QSYQ’s gene targets. The transcriptome revealed 18,458 genes expressed in the QSYQ group, and 18,349 in the model group (Figure 3A). Out of these, 307 differentially expressed genes (DEGs) were significant, with fold changes ≥1 and P-values ≤0.05, comprising 133 upregulated and 174 downregulated genes (Figure 3B). A hierarchical cluster analysis of these DEGs is depicted in Figure 3C.

**FIGURE 4:**
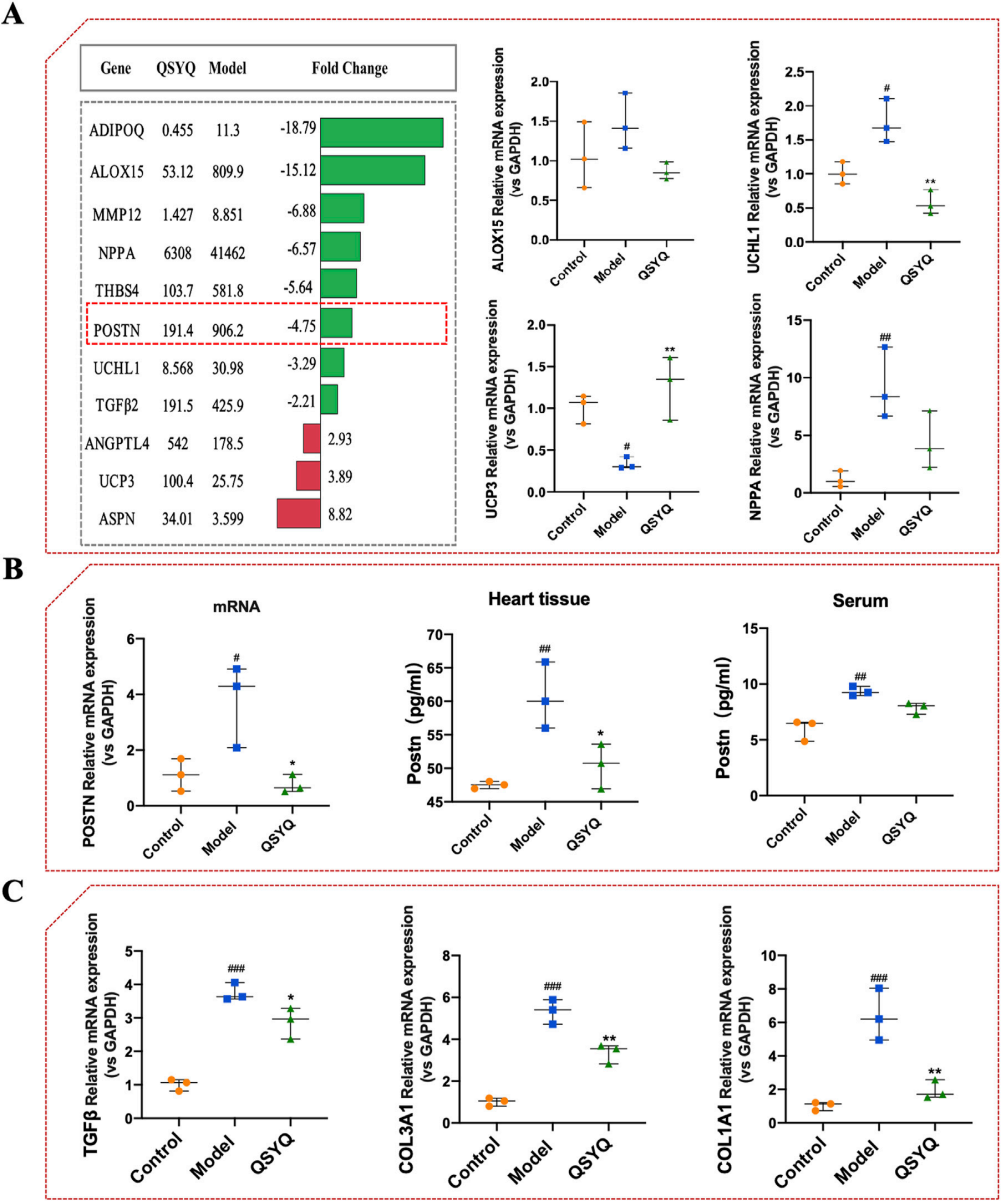
Identification and validation of key differentially expressed genes by QSYQ in Dahl hypertensive rat hearts. **(A)** Most significantly regulated genes by QSYQ in Dahl hypertensive rat hearts revealed by transcriptome analysis and RT-qPCR validation of Nppa, Uchl1, Ucp3, and Alox15 (*n* = 3). **(B)** Quantitative assessment of the POSTN mRNA and periostin protein expression in heart tissue and rat serum using PR-qPCR and ELISA. **(C)** Relative expression of heart TGF-β, Col1a1, and Col3a1 mRNAs evaluated by RT-qPCR (*n* = 3). Data were expressed as mean ± SEM. ^#^ *P* < 0.05, ^##^ *P* < 0.01, ^###^ *P* < 0.001 vs. Control; ^*^ *P* < 0.05, ^**^ *P* < 0.01 vs. Model.

Network pharmacology analysis of the transcriptome results by IPA revealed that the top 10 functions affected by QSYQ, ranked in descending order of -log (p-value) scores, are inflammatory response, organismal injury and abnormalities, organismal development, connective tissue disorders, skeletal and muscular disorders, cellular assembly and organization, hereditary disorder, developmental disorder, connective tissue development and function and tissue morphology (Figure 3D). As the inflammatory response and organismal injury and abnormalities ranked number one and two in the -log (p-value) score, 47 targets related to inflammation of organs were selected from the inflammation response (Figure 4E) and 19 targets correlated with abnormality of heart ventricle were selected from organismal injury and abnormalities regulated by QSYQ (Figure 4F). The close relationship involved in multiple shared and unique targets between inflammatory response and organismal injury and abnormalities was also verified in the network (Figure 4G).

### 3.4 Verification of DEGs and the effect of QSYQ on fibrosis target in Dahl hypertensive rats

IPA Core analysis identified eight genes—Adipoq, Igf2, Itgb3, POSTN, Mme, Gabra4, Adra2c, and Vip—as shared targets in Dahl rat hearts influenced by both inflammation and left ventricular abnormalities (Figure 3G). By combining transcriptome date and liture review, these genes are suggested as key regulators affected by QSYQ in hypertensive hearts (Figure 4A). RT-qPCR confirmed QSYQ significantly modulated Nppa, Uchl1, and Ucp3 expression (Figure 4A). Notably, both network pharmacology and transcriptome analyses placed POSTN (encoding periostin protein) at the interface of the inflammatory response and organismal injury and abnormalities (Figures 3, 4A). Thus, these results suggested that POSTN might be a key target of QSYQ in Dahl hypertensive rat hearts, supporting the reported role of periostin as an important marker in hypertensive cardiac fibrosis. To verify the RNA sequencing data, RT-qPCR and ELISA tests were conducted. Results indicated a rise in POSTN mRNA levels in the model group compared to the control (1.11 ± 0.58 vs. 3.76 ± 1.49, as displayed in Figure 4B). Similarly, periostin protein levels were notably higher in the heart (47.51 ± 0.53 vs. 60.62 ± 4.96) and serum (5.97 ± 0.96 vs. 9.34 ± 0.42) of hypertensive model rats than in controls. QSYQ markedly reduced the mRNA expression of POSTN (0.76 ± 0.32 vs. 3.76 ± 1.49), as well as the periostin protein expression in both the heart (50.44 ± 3.34 vs. 60.62 ± 4.96) and serum (7.88 ± 0.51 vs. 9.34 ± 0.42). Additionally, fibrosis indicators TGF-β, Col1a1 and Col3a1 mRNA showed a significant increase in the hypertensive heart of the model rats compared to controls, indicating an increase in fibrotic activity (1.01 ± 0.177 vs. 3.75 ± 0.27 for TGF-β, 1.02 ± 0.25 vs. 6.39 ± 1.55 for Col1a1, and 1.01 ± 0.19 vs. 5.34 ± 0.59 for Col3a1, respectively). QSYQ reduced the mRNA expression of TGF-β, Col1a1 and Col3a1 (2.87 ± 0.46 vs. 3.75 ± 0.27 for TGF-β, 1.95 ± 0.56 vs. 6.39 ± 1.56 for Col1a1, and 3.36 ± 0.46 vs. 5.34 ± 0.59 for Col3a1, respectively (Figure 4C). The findings suggest that QSYQ mitigates hypertensive cardiac damage in Dahl rats by modulating periostin and downstream fibrotic genes such as TGF-β, Col1a1, and Col3a1.

### 3.5 QSYQ reduced Ang II-induced hypertrophy and vimentin expression in cardiac spheroid model

**FIGURE 5:**
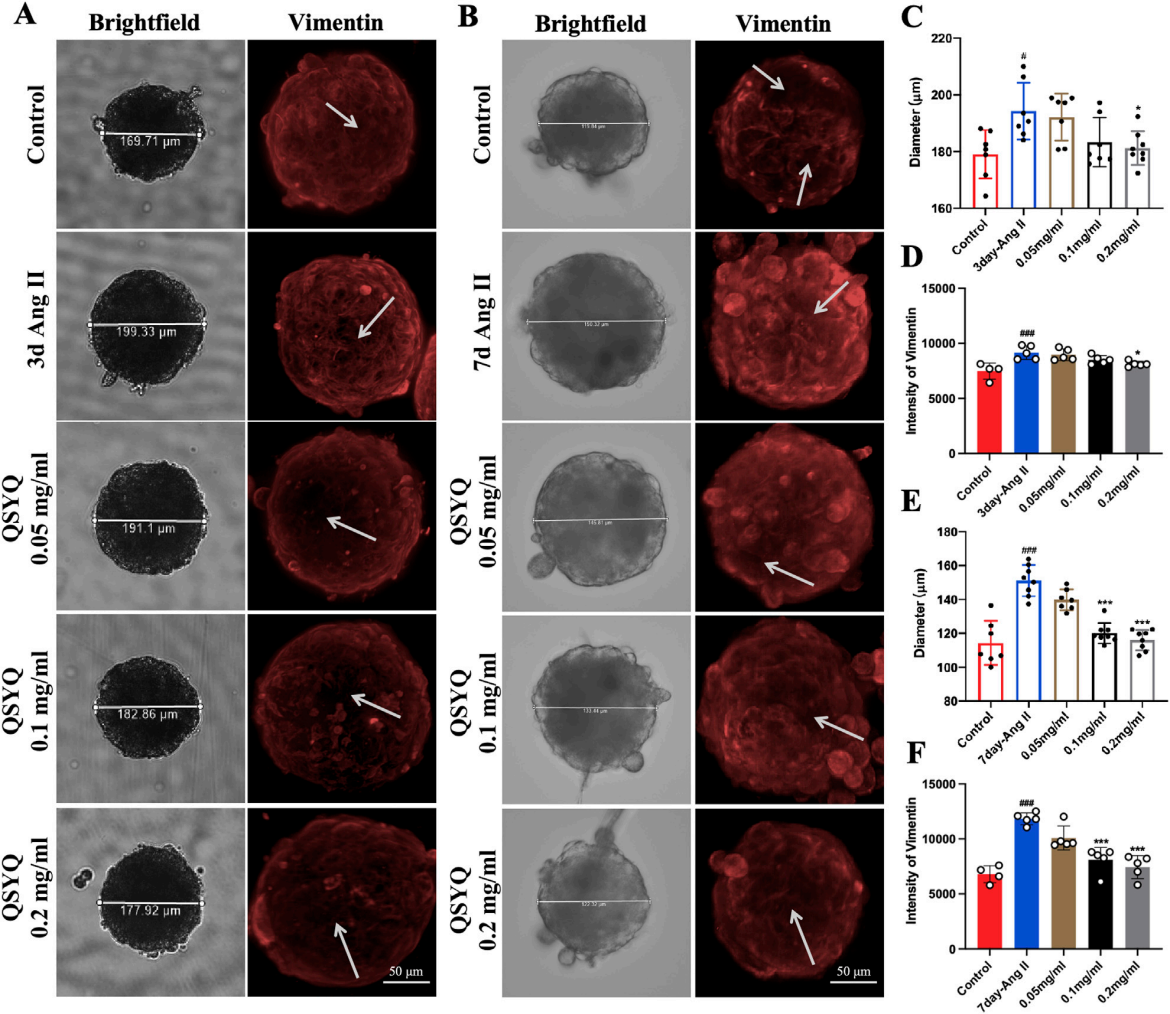
QSYQ reduced Ang II-inducted cardiac hypertrophy and Vimentin expression in cardiac spheroids. **(A)** Representative images of brightfield and immunofluorescence staining of Ang II-treated cardiac spheroid for 3 days. **(B)** Representative images of brightfield and immunofluorescence staining of Ang II-treated cardiac spheroid for 7 days. The white arrows indicate expression of fibroblast in cardiac spheroids. **(C)** Quantification of the cardiac spheroid diameters at day 3 of Ang II-treatment. **(D)** Quantification of the vimentin immunofluorescence intensity at day 3 of Ang II-treatment. **(E)** Quantification of the cardiac spheroid diameters at day 7 of Ang II-treatment. **(F)** Quantification of the vimentin immunofluorescence intensity at day 7 of Ang II-treatment. Data were expressed as mean ± SEM. ^#^ *P* < 0.05, ^###^ *P* < 0.001 vs. Control; ^*^ *P* < 0.05, ^***^ *P* < 0.001 vs. Ang II.

To further evaluate the protective effect of QSYQ on cardiac hypertrophy *in vitro*, we established an Ang II-induced hypertrophy model in cardiac spheroids. Brightfield and immunofluorescence staining showed that after Ang II treatment for 3 days, the diameter of the cardiac spheroid increased from 179.09 ± 8.52 to 194.26 ± 10.03, proliferation of cardiac fibroblasts and the expression of vimentin increased from 7484.5 ± 732.33 to 9016 ± 608.20 (Figures 5A,C). After Ang II treatment for 7 days, the diameter of the cardiac spheroid increased from 114.35 ± 13.04 to 149.79 ± 8.99 and the expression of vimentin increased from 6794 ± 777.16 to 11838 ± 616.81 (Figures 5B,D). On the other hand, QSYQ treatment dose-dependently decreased the diameter of spheroids and the vimentin expression. Specifically, at day 3, only 0.2 mg/mL QSYQ slightly reduced the diameter of spheroids to 181.47 ± 6.36, while 0.05, and 0.1 mg/mL QSYQ had no effect (Figures 5A,C). At day 7, 0.1 and 0.2 mg/mL QSYQ reduced the diameter of spheroids to 183.34 ± 8.69 and 181.47 ± 6.36, respectively (Figures 5B,D). Similarly, at day 3, only 0.2 mg/mL QSYQ reduced the vimentin expression to 8127 ± 284.84 (Figures 5A,C). At day 7, both 0.1 and 0.2 mg/mL QSYQ reduced the diameter of spheroids to 118.27 ± 3.17, and 115.17 ± 5.92 (Figures 5B,E) and the vimentin expression to 8595.5 ± 259.96, and 7349.25 ± 1184.64 (Figures 5B,F).

### 3.6 QSYQ alleviated Ang II-induced fibrosis in a cardiac spheroid model

**FIGURE 6:**
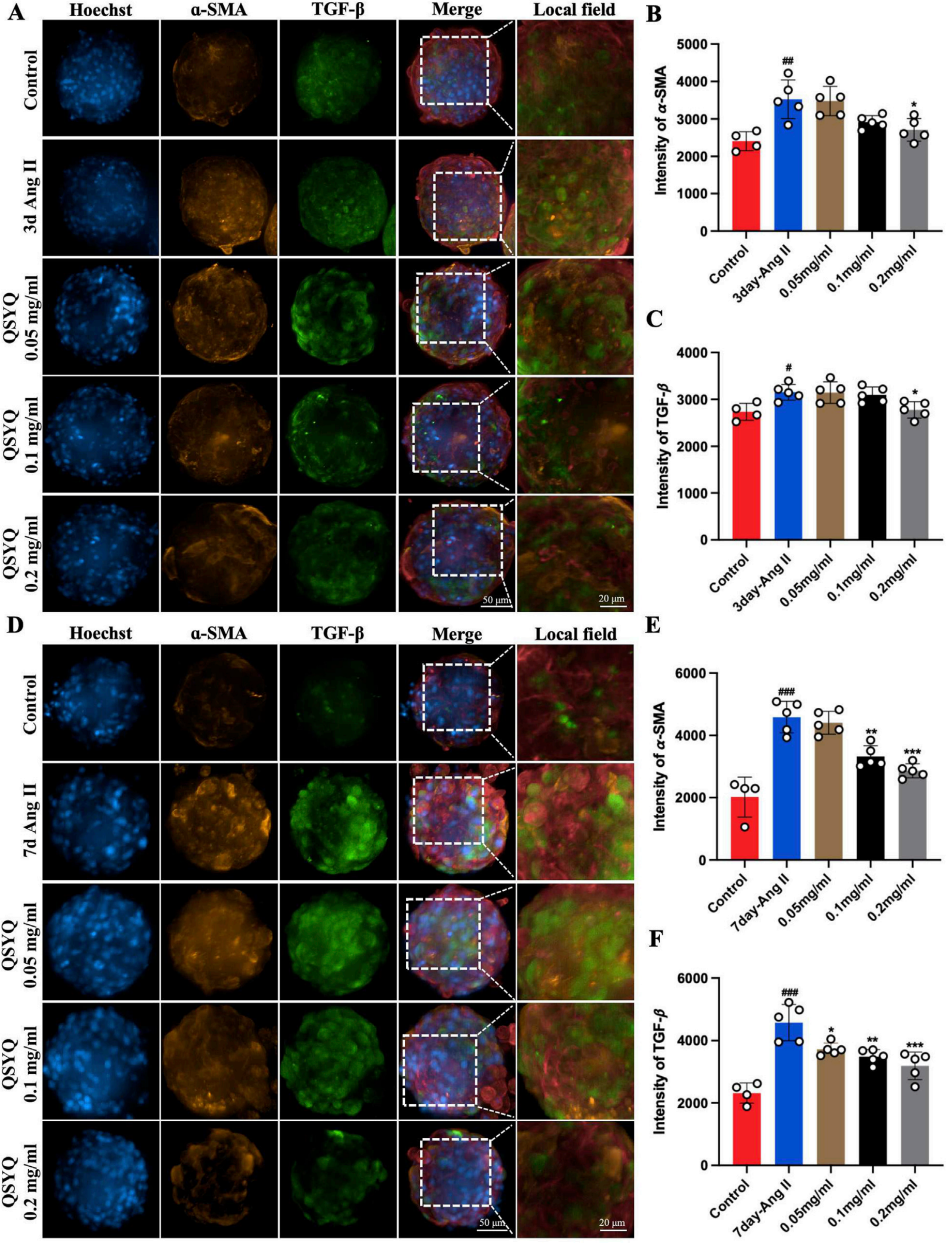
QSYQ alleviated Ang II-inducted fibrosis in cardiac spheroids. **(A)** Representative images of immunofluorescence staining of Ang II-treated cardiac spheroid for 3 days. (from left to right) Hoechst, *α*-SMA, TGF-β, merge and its enlarged local field. **(B,C)** Quantification of the fluorescence intensity of *α*-SMA **(B)** and TGF-β **(C)** at 3 days of Ang II treatment in each group. **(D)** Representative images of immunofluorescence staining of Ang II-treated cardiac spheroid for 7 days. (from left to right) Hoechst, *α*-SMA, TGF-β, merge and its enlarged local field. **(E,F)** Quantification of the fluorescence intensity of *α*-SMA **(E)** and TGF-β **(F)** at 7 days Ang II-treatment cardiac spheroid in each group. Data were expressed as mean ± SEM. ^#^ *P* < 0.05, ^##^ *P* < 0.01, ^###^ *P* < 0.001 vs. Control; ^*^ *P* < 0.05, ^**^ *P* < 0.01, ^*^ *P* < 0.001 vs. Ang II.

It is increasingly recognized that Ang II causes hypertension and contribute to fibrosis associated with hypertension-related heart disease (Failer et al., 2022). Cardiac organoids treated for 3 and 7 days with Ang II were used to assess the cardiac fibrosis process and to evaluate the protective effect of QSYQ *in vitro*. Immunofluorescence staining results showed that compared to that of the control, 3 and 7 days of Ang II treatment increased *α*-SMA (3351.25 ± 388.84, and 4549.75 ± 577.22, Figures 6A,B,D,E) and TGF-β expression (3208.5 ± 134.19, and 4729.75 ± 540.86, Figures 6A,C,D,F) in cardiac spheroids. 0.2 mg/mL QSYQ treatment after 3 days of Ang II decreased *α*-SMA (2734.25 ± 342.32, Figures 6A,B) and TGF-β expression (2729.75 ± 163.48, Figures 6A,C). Meanwhile, QSYQ treatment after 7 days of Ang II dose-dependently decreased *α*-SMA (4518 ± 310.17 at 0.05 mg/mL, 3368.5 ± 384.30 at 0.1 mg/mL and 2935.75 ± 190.37 at 0.2 mg/mL, Figures 6D,E) and TGF-β expression (3720.75 ± 232.28 at 0.05 mg/mL, 3429.5 ± 223.36 at 0.1 mg/mL and 3138 ± 487.02 at 0.2 mg/mL, Figures 6D,F). Overall, these data suggest that 7 days of Ang II treatment of cardiac spheroids is a preferred cardiac fibrosis model in which QSYQ has significant alleviating effect. Therefore, the 7-day model was chosen for our follow-up experiments.

### 3.7 QSYQ reduced pathological development of fibrosis and collagen deposition in cardiac spheroids induced by Ang II

**FIGURE 7:**
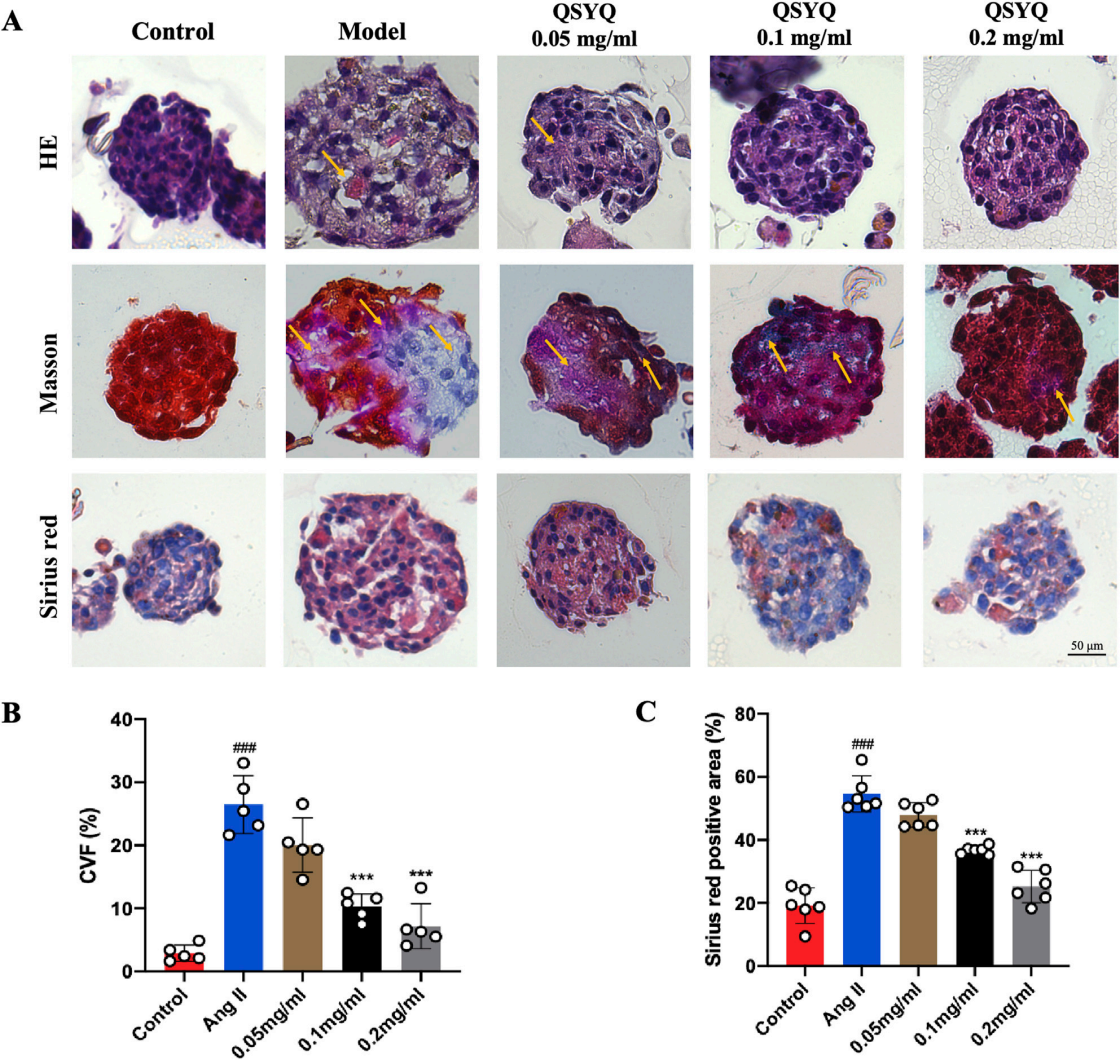
QSYQ inhibited fibrotic development and collagen deposition in Ang II-induced cardiac spheroids. **(A)** Representative images of H&E, Masson and Sirius red staining of cardiac spheroids in each group (*n* = 5–6). The yellow arrows indicate pathological changes, the area of fibrosis and the area of myocardial interstitial fibrosis in cardiac spheroids. **(B)** Quantification of collagen volume fraction of cardiac spheroid in each group (*n* = 5). **(C)** Quantification of Sirius red positive area of the cardiac spheroid in each group (*n* = 6). Data were expressed as mean ± SEM. ^###^ *P* < 0.001 vs. Control; ^*^ *P* < 0.001 vs. Ang II.

H&E, Masson and Sirius red staining were performed to further evaluate the anti-myocardial fibrosis effect of QSYQ. The Ang II-induced pathological development of fibrosis in the cardiac spheroids is profound, as indicated by increased cell space and necrosis in H&E staining and increased ECM deposition in Masson and Sirius red (Figures 7A–C). QSYQ dose-dependently reversed the pathological structural changes of cardiac spheroids induced by Ang II, reducing CVF to 21.42 ± 3.46 at 0.05 mg/mL, 10.17 ± 2.28 at 0.1 mg/mL and 7.58 ± 3.98 at 0.2 mg/mL, Figure 7B. QSYQ also dose-dependently reversed the fibrotic pathological indicators induced by Ang II, reducing Masson and Sirius red staining area to 47.54 ± 4.15 at 0.05 mg/mL, 37.06 ± 1.12 at 0.1 mg/mL and 25.94 ± 5.46 at 0.2 mg/mL, Figure 7C.

### 3.8 QSYQ reduced periostin protein expression in cardiac spheroids and CFs

**FIGURE 8:**
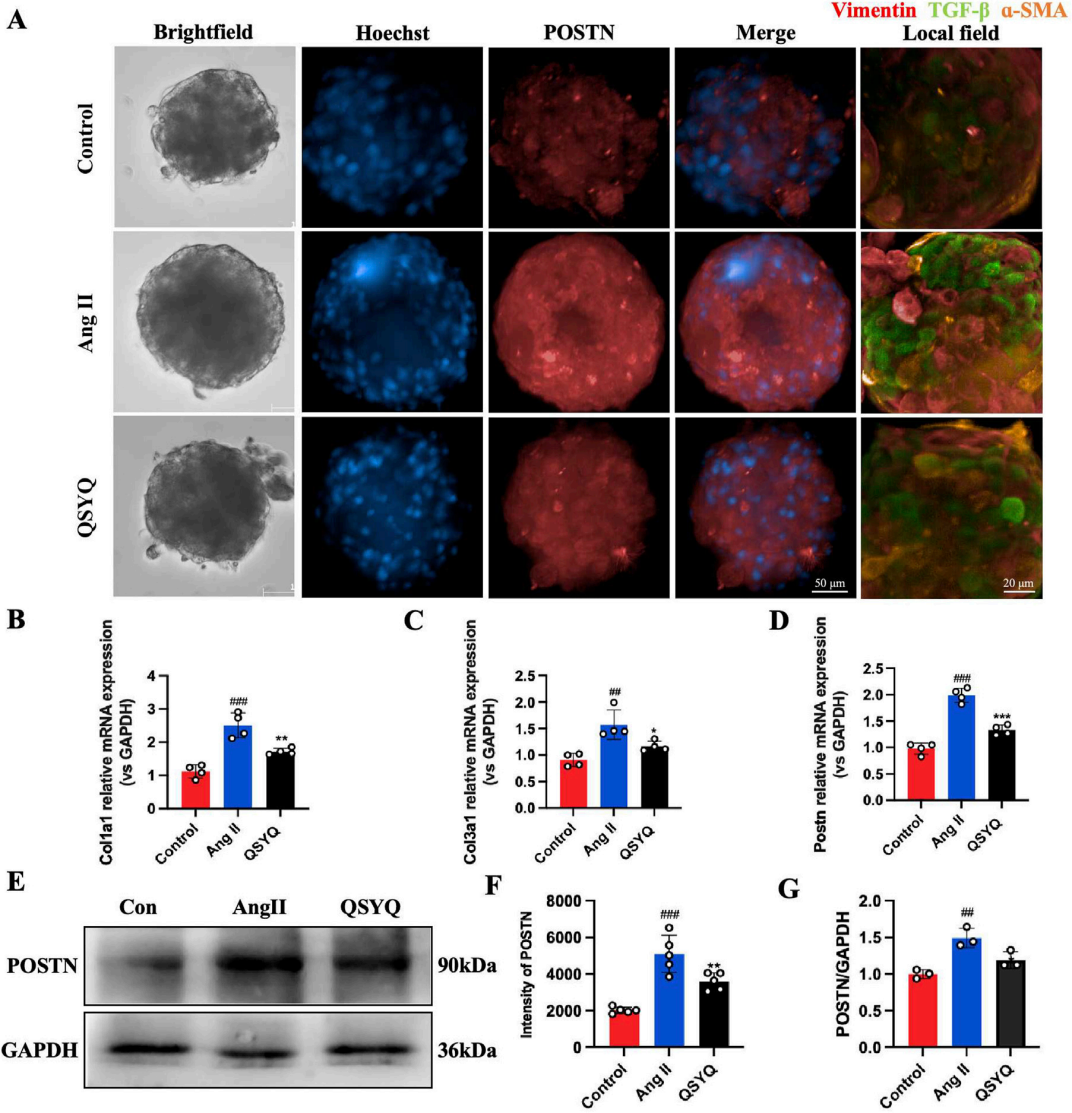
QSYQ inhibited the expression of POSTN, Vimentin, *α*-SMA and TGF-β in cardiac spheroids and Col1a1 and Col3a1 in CFs cells after Ang II treatment. **(A)** Representative images of control, Ang II, and Ang II + QSYQ-treated cardiac spheroids. **(B–D)** RT-PCR verification of QSYQ regulated fibrosis genes in Col1A1, Col3a1 and POSTN (*n* = 4). **(E)** Representative blots of POSTN in the control, Ang II and QSYQ (0.1 mg/mL) groups (*n* = 3). **(F)** Quantification of the immunofluorescence intensity of POSTN in each group (*n* = 5). **(G)** Quantification of expression levels of POSTN protein (*n* = 3). Values were expressed as mean ± SD. ^##^ *P* < 0.01, ^###^ *P* < 0.001 vs. Control; ^*^ *P* < 0.05, ^**^ *P* < 0.01, ^*^ *P* < 0.001 vs. Ang II.

The expression of periostin (POSTN) protein in Ang II-induced cardiac spheroid and CF cell models were examined using IF and WB. Ang II-induced cardiac spheroids significantly increased the intensity of POSTN, compared to that in control (2017 ± 169.55 vs. 5099.4 ± 1014.40, Figures 8A,F). QSYQ markedly reversed the overexpression of POSTN protein in cardiac spheroids caused by Ang II-induced cardiac fibrosis (3589.8 ± 472.78 vs. 5099.4 ± 1014.40, Figures 8A,F). Similar to that of WB and RT-qPCR analysis (Figures 8D,E), QSYQ at a dose of 0.1 mg/mL significantly decreased the POSTN (1.19 ± 0.12), and effectively protected CF cells from Ang II-induced fibrosis (1.19 ± 0.12 vs. 1.49 ± 0.13, Figure 8G). At the same time, QSYQ reduced the intensity of Vimentin, *α*-SMA and TGF-β in cardiac spheroids (Figure 8A) and Col1a1, Col3a1 and Postn mRNA expression (1.72 ± 0.10 vs. 2.50 ± 0.38, 1.17 ± 0.10 vs. 1.57 ± 0.28, 1.33 ± 0.10 vs. 1.99 ± 0.14) in CF cells, compared with Ang II group. These results were consistent with the results of the *in vivo* experiments, and suggested that POSTN may be a key target to protect cardiac spheroids and CF cells from Ang II-induced fibrosis by QSYQ.

## 4 Discussion

**FIGURE 9:**
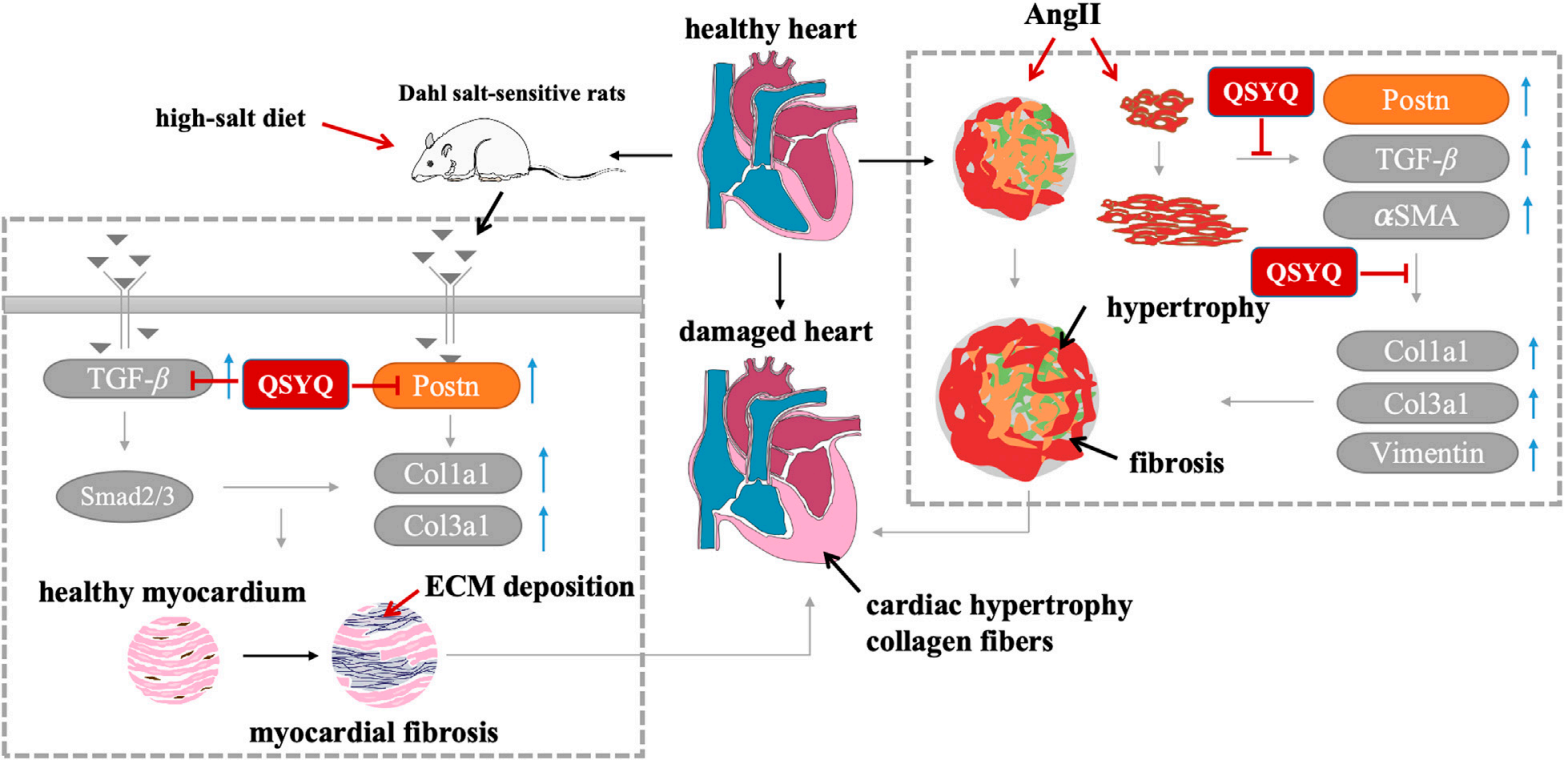
A schematic diagram of the mechanism. QSYQ alleviates cardiac fibrosis in Dahl salt-sensitive hypertensive rat hearts, 3D cardiac spheroids and 2D cardiac fibroblasts via inhibiting the POSTN.

We have made three new findings in this study. First, *in vivo* study using Dahl hypertension rats demonstrated that a long-term high salt diet led to severe heart hypertrophy and fibrosis. QSYQ significantly improved cardiac function, attenuated myocardial hypertrophy and fibrosis. Second, we showed that QSYQ protects against hypertensive cardiac fibrosis and heart damage at least in part by regulating multiple signaling pathway activator periostin. Third, using Ang II treatment of cardiac spheroids induced hypertrophy and fibrosis *in vitro*. QSYQ reversed hypertrophic and fibrotic phenotypes and downregulated the expression of periostin and related hypertrophic and fibrotic genes (Figure 9).

Dahl salt-sensitive (Dahl SS) rat is a strain of hypertensive animals that develop systemic hypertension based on the sodium content provided in the diet (Dahl et al., 1962; Zicha et al., 2012). Dahl SS rats fed a high-salt diet increased systolic blood pressure of more than 200 mmHg due to a combination of pressure and volume overload and a high-salt diet for five to 6 weeks led to compensatory left ventricular hypertrophy (Tran et al., 2016). Dahl SS rats developed heart failure after 10–12 weeks of a high-salt diet, accompanied by left ventricular dilatation and systolic dysfunction (Iwanaga et al., 1998). Our study confirms these rats suffer from diastolic dysfunction after 9 weeks on a high-salt diet (Figure 1). QSYQ treatment notably improves diastolic function and reduces cardiac hypertrophy and fibrosis in these rats (Figure 2), suggesting its effectiveness in mitigating cardiac damage from salt-sensitive hypertension.

Recent studies have found that Traditional Chinese medicines and other herbal medicines protect against hypertensive heart damage in Dahl salt-sensitive hypertensive rats. Guizhi Decoction, Guizhi Decoction, comprising ingredients like Guizhi and ginger, improves myocardial fibrosis by modulating NGF and LIF levels, slowing heart failure progression (Wang et al., 2020). Xiao-Qing-Long-Tang, with components such as ephedra and licorice, maintains cardiac function by correcting sympathetic nervous system imbalances (Li et al., 2020). Puerarin improves vascular insulin resistance and cardiovascular remodeling in salt-sensitive hypertension (Tan et al., 2017). Investigating QSYQ’s effects, RNA sequencing revealed 307 differentially expressed genes associated with inflammation and organ injury (Figure 3). RT-qPCR confirmed the significant upregulation of POSTN, a gene closely linked to hypertensive heart damage (Figure 3). Thus, integrated analyses suggest that hypertensive conditions increase POSTN expression in cardiac tissue.

Cardiac fibrosis is a common and key pathological process leading to heart failure in various heart diseases (Frangogiannis, 2021). Our results provided an *in vitro* 3D model of hypertensive heart disease. Early studies have shown that Ang II not only raises blood pressure but also induces cardiac hypertrophy and myocardial expansion (De Mello and Danser, 2000; Sadoshima and Izumo, 1993; Schlüter and Wenzel, 2008). It is interesting that Ang II has been shown to stimulate significant growth in other cell types, such as fibroblasts, in addition to the hypertrophy of cardiomyocytes (Kim and Iwao, 2000; Verma et al., 2021), which ultimately leads to fibrosis. We have developed a 3D spheroid model that mimics hypertensive heart disease *in vitro*, displaying hypertrophy and fibrosis when treated with Ang II (Figures 5–7). This model effectively replicates the key characteristics of heart fibrosis and allows for the study of intercellular signaling relevant to the disease.

A large amount of evidence indicates that POSTN plays a vital role in coronary artery disease and cardiac fibrosis caused by hypertension (Wu et al., 2016; Zhao et al., 2014). As an extracellular matrix (ECM) protein, Periostin is prominently expressed in fibrotic tissue (Mael-Ainin et al., 2014; Okamoto et al., 2011; Wallace et al., 2014), influenced by TGF-β pathways in mesenchymal cells, a key factor in fibrosis (Ashley et al., 2017; Egbert et al., 2014). High salt intake by Dahl SS rats leads to hypertension, ventricular hypertrophy, and heart failure (Tran et al., 2016). Myocardial fibrosis in these rats is linked to TGFβ activation, and TGFβ1 can induce POSTN expression in cardiac and vascular cells (Li et al., 2006; Snider et al., 2008). POSTN also interacts with ECM components like fibronectin, affecting tissue biomechanics (Kii et al., 2010). Ang II increases arterial pressure by inducing vasoconstriction and promoting sodium and water reuptake in the renal tubules (Failer et al., 2022), promotes systemic arteriole constriction, causes hypertension by binding to Ang II type I receptors, and contributes to fibrosis in hypertension-related heart disease (Funck et al., 1997; Maning et al., 2017). Therefore, *in vitro* experiments were performed in this study using cardiac organoids treated with Ang II to investigate the effect of POSTN on hypertensive cardiac fibrosis (Figure 6). Studies have shown that the expression of POSTN is increased in hypertensive hearts (Figure 8). Inhibiting the expression of POSTN may be a promising method to attenuate the heart remodeling caused by hypertension (Wu et al., 2016). QSYQ was found to lower blood pressure (Du et al., 2021), reduce diastolic dysfunction and hypertrophy, and alleviate cardiac fibrosis in hypertensive rats, suggesting that QSYQ’s cardioprotective effects stem from lowering POSTN and TGF-β expression, inhibiting collagen synthesis, and reducing ECM formation, potentially inhibiting fibrosis progression. (Figures 4, 7, 8).

Our study indicates that QSYQ mitigates myocardial damage in salt-sensitive hypertensive conditions by modulating the expression of fibrotic genes such as POSTN and TGF-β. Nevertheless, further investigation is necessary due to: (1) differences between animal models and human pathology, limiting direct translation of findings to humans, (2) the need to identify which components of QSYQ crucially influence fibrosis pathways, and (3) the necessity to explore other key transcriptome genes, such as Uchl1 and Ucp3, for a comprehensive understanding of the multi-component and multi-targeted action of traditional Chinese medicine.

## 5 Conclusion

Our research showed that QSYQ effectively improves cardiac function and reduces hypertrophy and fibrosis in salt-sensitive hypertensive rats, at least in part due to its regulatory effect on the signaling activator periostin. This suggests QSYQ’s therapeutic promise for salt-sensitive hypertensive cardiac injury by targeting hypertrophic and fibrotic pathways.

## Data Availability

The datasets presented in this study can be found in online repositories. The names of the repository/repositories and accession number(s) can be found in the article/Supplementary Material.

## Abbreviations

Ang II, angiotensin II; CFs, Cardiac fibroblasts; CMs, cardiomyocytes; Dahl SS, Dahl salt-sensitive; DEGs, Differentially Expressed Genes; DMEM, Dulbecco’s modified eagle medium; ECM, extracellular matrix; ECs, endothelial cells; IF, Immunofluorescence; LV, left ventricle; SD, Sprague Dawley; POSTN, Periostin; QSYQ, Qishen Yiqi dripping pill; WB, Western blotting.
