# Supplementary material for: Qishen Yiqi alleviates periostin-mediated cardiac fibrosis and hypertrophy in Dahl hypertensive rat hearts and angiotensin II-induced cardiac organoids
Source: Front Pharmacol. 2025 Jun 18;16:1491582. doi: 10.3389/fphar.2025.1491582 (PMC12213470; doi:10.3389/fphar.2025.1491582)
Supplement: Supplementary file 1 [file DataSheet1.docx]

**Supplemental Information**

**Drugs and reagents**

Please refer to the Supplemental information for the Materials and Methods section. Qishen Yiqi (drug approval number: Z20030139; batch number: 20160604) was provided by Tasly Pharmaceutical Group Co, Ltd. It was prepared in the ratio of Astragalus: Salvia miltiorrhiza: Panax notoginseng: Jiangxiang oil=148.01:70.35:70.35:11.97 and dissolved in ultra-pure water to make 15mg/ml solution for the experiment. Hydrochlorothiazide (HCTZ) (drug approval number: H12020166; batch number: 1904009) was purchased from Tianjin Lisheng Pharmaceutical Group Co., Ltd., and was dissolved in ultrapure water to prepare a 2mg/ml solution. Lactate dehydrogenase (LDH) kit was purchased from Nanjing Jiancheng Institute of Bioengineering (A020-2, Nanjing, China). TRIzolTM reagent (15596026) was purchased from Ambion, USA. The reverse transcription kit was purchased from Roche, Germany（04897030001, Mannheim, Germany. Bestar™ qPCR MasterMix was purchased from DBI Company (2043, Beijing, China). POSTN ELLISA kit was purchased from Shanghai Zhuokai Biotechnology Co., Ltd (ZC-36167, Shanghai, China). M199 medium was purchased from Gibco (12340030, California, United States). Anti-vimentin antibody (ab24525), Anti-𝛼-SMA antibody (ab7817), Goat Anti-Mouse IgG H&L (Alexa Fluor® 594, ab150116), Donkey Anti-Rabbit IgG H&L (Alexa Fluor® 488, ab150073) and Goat Anti-Chicken [IgY](https://www.sciencedirect.com/topics/medicine-and-dentistry/immunoglobulin-y) H&L (Alexa Fluor® 647, ab150175) were purchased from Abcam (Cambridge, United Kingdom).  Anti-POSTN polyclonal antibody was purchased from Bioss (bs-4994R, Beijing, China). Anti-TGF-β antibody (21898-1-AP) was purchased from Proteintech Group, Inc. (Wuhan, China). Masson trichromatic staining kit (G1346) and Picro Sirius Red staining kit (PH1099) were purchased from Solarbio (Beijing, China).

**Animal grouping and drug treatment**

Dahl SS rats were adapted to a standard raising condition for one week. The control group was fed with normal salt diet while the model and treatment groups were fed with high-salt diet (8% NaCl) for five weeks. Afterwards, the high-salt fed animals were randomly divided into three groups, including the Model group (pure water, N=6), the QishenYiqi group (150mg/kg, QSYQ, N=6), the hydrochlorothiazide group (20mg/kg HCTZ, N=4). The administration lasted for four weeks once a day, the control group (pure water, 10 ml/kg, N=4) was given a normal salt diet and the other three groups were given high salt diet.

**Echocardiography**

After four weeks of drug administration, rats were anesthetized with isoflurane and cardiac function and morphology were evaluated by transthoracic echocardiography using an ultra-high resolution small animal ultrasound imaging system (Vevo 2100, VisualSonics, Toronto, ON, Canada). The left ventricular function was assessed in M-mode with the following indexes: left ventricular (LV) ejection fraction (EF), LV fractional shortening (FS), LV septal thickness at diastole (LVSd), LV septal thickness at systole (LVSs), LV posterior wall at diastole (LVPWd), LV posterior wall at systole (LVPWs) and LV mass. Left ventricular early diastolic peak blood flow / late diastolic peak blood flow (E/A) was evaluated in PW Doppler mode.

**Measurement of pathological parameters**

Heart tissue was fixed in 4% paraformaldehyde for more than 48 hours and then dehydrated and embedded. Tissue blocks were cut into 5μm thick coronal slices using a manual slicer (HM355S, Thermo., Ltd., USA). Heart tissue slices were stained with H&E for histopathology, with Masson trichrome kit (Solarbio, China, G1346) for cardiac fibrosis, and with Picro Sirius Red Stain Kit (Solarbio, China, PH1099) for collagen deposition. The slices were sealed with an automatic sealer (ClearVue, Thermo, Ltd., USA) and images analyzed with an automatic quantitative pathological imaging system (Vectra 3, PerkinElmer, USA) . Five randomly selected fields were viewed and photographed with a 200x lens. The positive signals of each slice were quantified using ImagePro Plus 6.0 software (National Institutes of Health, Bethesda, MD, United States).

**Transcriptome sequencing**

Total RNA was extracted from heart tissue and its purity and integrity were detected by agarose gel electrophoresis and Nano Photometer spectrophotometer. The Sequencing libraries were constructed using Illumina's NEBNext ® UltraTM RNA Library Prep Kit and its quality determined using a Qubit2.0 fluorometer and Agilent 2100 bioanalyzer. Adapters, reads containing N and low-quality reads in the raw data of 150 bp paired-end reads obtained by Illumina sequencing were removed. Based on the length of the gene, FPKM of each gene were calculated and the reads were mapped to that gene. DESeq2 software (1.16.1) was used to analyze the differential expression between the model and QSYQ groups. Log2FoldChange ≥1 and P value ≤0.05 were set as thresholds for significant differential expression.

**Core Analysis of Differentially Expressed Genes (DEGs)**

The core analysis of RNA-Seq gene expression data was performed using ® Ingenuity Pathway Analysis (IPA), selecting the DEGs with fold change ≥ 2, P-value ≤ 0.05. The IPA Knowledge Base was set as the reference set when carrying out data analysis. Excluding endogenous chemicals, Direct and indirect relationships. Significance was measured by the ratio of the number of molecules mapped to the dataset in the pathway divided by the total number of genes in the pathway and the calculated P-value Fisher's exact test.

**Real time reverse transcription polymerase chain reaction (RT-PCR) assay**

TRIzolTM reagent was used to extract total RNA from rat heart tissues and cardiac fibroblast according to the manufacturer's instructions. Transcriptor First Strand cDNA Synthesis Kit (Roche, Mannheim, Germany) was used to reverse transcribe the RNA into cDNA. According to the instructions, the cDNA, Bestar™ qPCR Master Mix (2043, DBI Bioscience, Shanghai, China) and primers (Shanghai, China) were mixed into the PCR strip tubes. Then the real-time PCR system (LightCycler®480, Roche (Germany)) was used to detect the mRNA expression level of each sample. In this experiment, the target genes to be quantified include *Nppa*, *Uchl1*, *Ucp3*, *Alox15*, *POSTN*, *Tgf-β*, *Col1a1* and *Col3a1*. Glyceraldehyde-3-phosphate dehydrogenase (Gapdh) was used for standardization, and the primer sequences were described in Table 1.

Table 1. Primer sequences for qRT-PCR.

| Primer name | Primer sequence (5’-3’) |
| --- | --- |
| *Nppa forward* | TTCTCCATCACCAAGGGCTTCTTC |
| *Nppa reverse* | CCAGGTGGTCTAGCAGGTTCTTG |
| *Uchl1 forward* | CCGATGGAGATTAACCCCGAGATG |
| *Uchl1 reverse* | GCAGGCAGGAGATGGCACTG |
| *Ucp3 forward* | CATCGCCAGGGAAGAAGGAGTC |
| *Ucp3 reverse* | GTCAGTGAACAGGTGAGAGTCCAG |
| *Alox15 forward* | ATGACTTGGCTGAGCGAGGAC |
| *Alox15 reverse* | GTCGGTCTTGTAGTGGAGATTGAAC |
| *POSTN forward* | GAGCCACGACCACTCAACACTATTC |
| *POSTN reverse* | TGTTCTCTAGTCCTCTGCGGATGTC |
| *Tgf-β forward* | GACCGCAACAACGCAATCTATGAC |
| *Tgf-β reverse* | CTGGCACTGCTTCCCGAATGTC |
| *Col1a1 forward* | TGTTGGTCCTGCTGGCAAGAATG |
| *Col1a1 reverse* | GTCACCTTGTTCGCCTGTCTCAC |
| *Col3a1 forward* | AGTCGGAGGAATGGGTGGCTATC |
| *Col3a1 reverse* | CAGGAGATCCAGGATGTCCAGAGG |

**ELISA**

POSTN was determined from heart tissue and serum using Elisa kit (ZC-36167, Shanghai ZCIBIO Technology Co., Ltd.), the expression level of POSTN were measured with a Tecan Spark microplate reader (Tecan, Groedig, Austria) at a wavelength of 450nm. The standard curve from the kit, which included standards at gradient concentrations, was used to compute the protein concentration of POSTN. The OD value is brought into the formula of the standard curve to obtain the protein concentration of POSTN in serum and tissue.

**Establishment of cardiac fibrosis model and drug administration**

Cardiac cell suspensions were cultured in M199 medium with 5% FBS till 3D cardiac spheroid formed. Then, Ang II was given to induce cardiac spheroid fibrosis as described previously(Ma et al., 2023; Wang et al., 2018). In QSYQ study, the cardiac spheroids were divided into 5 groups: Control with normal growth medium, Model (Cardiac spheroids were treated with 0.1 μM Ang II), QSYQ doses (Cardiac spheroids were cultured in M199 medium containing 0.05 μM, 0.1 μM and 0.2 μM QSYQ and 0.1 μM Ang II for 3 days and 7 days respectively).

**Immunofluorescence (IF)**

Immunofluorescence of cardiac spheroid was performed as previously described(Fan et al., 2023). The cardiac spheroids were fixed in 4% paraformaldehyde solution for 30 min. After perforating with 0.5% Triton for 30 min, cardiac spheroids were washed, blocked with 5% BSA for 90 min, incubated with the indicated primary antibody (vimentin, 1:200; TGF-β, 1:300; 𝛼-SMA, 1:300; and POSTN, 1:200) overnight 4˚C, washed with PBST and then incubated with the corresponding secondary antibody (Alexa Fluor 488, 1:1000; Alexa Fluor 594, 1:1000; and Alexa Fluor 647, 1:1000) and Hoechst 3342 at room temperature for 2 h in the dark, and washed with PBST and PBS respectively. Finally, the expression levels of the target proteins were determined by measuring the fluorescence intensity using an Operetta high content analysis system (Vectra 3, PerkinElmer, Waltham, MA, United States).

**Histology**

As previously described(Fan et al., 2023), sections of cardiac organoids were fixed in 4% paraformaldehyde for 1 h, and then dehydrated in ethanol, ethanol/xylene, xylene, xylene/paraffin and paraffin solution respectively. The paraffin block containing the spheroids was cut into 4µm slices with a manual microtome (HM355S, Thermo Fisher Scientific, Waltham, MA, United States), and kept until use after the microsphere had been embedded using an embedding machine.

For sections of rat heart, heart tissue was dehydrated using an automatic dehydrator (Excelsior, Thermo Fisher Scientific, Ltd., Waltham, United States), embedded in paraffin, and fixed in 4% paraformaldehyde. A manual slicer (HM355S, Thermo Fisher Scientific, Ltd.) was then used to manually cut the implanted tissue into-5µm thick slices, as previously described(Xiao et al., 2022). The slices were then put into an automatic slice stainer for H&E staining (Gemini, Thermo Fisher Scientific, Ltd.). To evaluate the fibrosis of cardiac spheroids and heart tissue, collagen fibers and deposition were also stained using a Masson trichromatic staining kit (G1346, Solarbio, Beijing, China) and Picro Sirius Red staining kit (PH1099, Beijing, China). Finally, using an automated quantitative pathology imaging system (Vectra 3, PerkinElmer), the cardiac microspheres and mouse myocardial tissue were imaged and quantified.

**Western blotting (WB)**

Western blotting was performed as described previously(Huang et al., 2020). The CFs were lysed with high-efficiency RIPA cell lysate and centrifuged, and a BCA kit was used to assess the protein content in the supernatant. SDS-polyacrylamide gel electrophoresis was used to separate the proteins, which were transferred to a PVDF membrane. After blocking with 5% skim milk powder, the membranes were treated with the primary antibody (anti-POSTN, 1:1000) overnight at 4˚C, washed and then incubated for two hours with the appropriate secondary antibody. The protein bands were developed using BCL luminous solution, visualized using gel imaging equipment (BOX F3, Syngene, England) and quantified using ImageJ software (National institutes of Health, Bethesda, Maryland, USA).

Fan, S., Xiao, G., Ni, J., Zhao, Y., Du, H., Liang, Y., Lv, M., He, S., Fan, G., Zhu, Y., 2023. Guanxinning injection ameliorates cardiac remodeling in HF mouse and 3D heart spheroid models via p38/FOS/MMP1-mediated inhibition of myocardial hypertrophy and fibrosis. Biomed Pharmacother 162, 114642.

Huang, Y., Zhang, K., Jiang, M., Ni, J., Chen, J., Li, L., Deng, J., Zhu, Y., Mao, J., Gao, X., Fan, G., 2020. Regulation of energy metabolism by combination therapy attenuates cardiac metabolic remodeling in heart failure. Int J Biol Sci 16, 3133-3148.

Ma, Z.G., Yuan, Y.P., Fan, D., Zhang, X., Teng, T., Song, P., Kong, C.Y., Hu, C., Wei, W.Y., Tang, Q.Z., 2023. IRX2 regulates angiotensin II-induced cardiac fibrosis by transcriptionally activating EGR1 in male mice. Nat Commun 14, 4967.

Wang, L., Zhang, Y.L., Lin, Q.Y., Liu, Y., Guan, X.M., Ma, X.L., Cao, H.J., Liu, Y., Bai, J., Xia, Y.L., Du, J., Li, H.H., 2018. CXCL1-CXCR2 axis mediates angiotensin II-induced cardiac hypertrophy and remodelling through regulation of monocyte infiltration. Eur Heart J 39, 1818-1831.

Xiao, G., Liu, J., Wang, H., He, S., Liu, J., Fan, G., Lyu, M., Zhu, Y., 2022. CXCR1 and its downstream NF-κB inflammation signaling pathway as a key target of Guanxinning injection for myocardial ischemia/reperfusion injury. Front Immunol 13, 1007341.


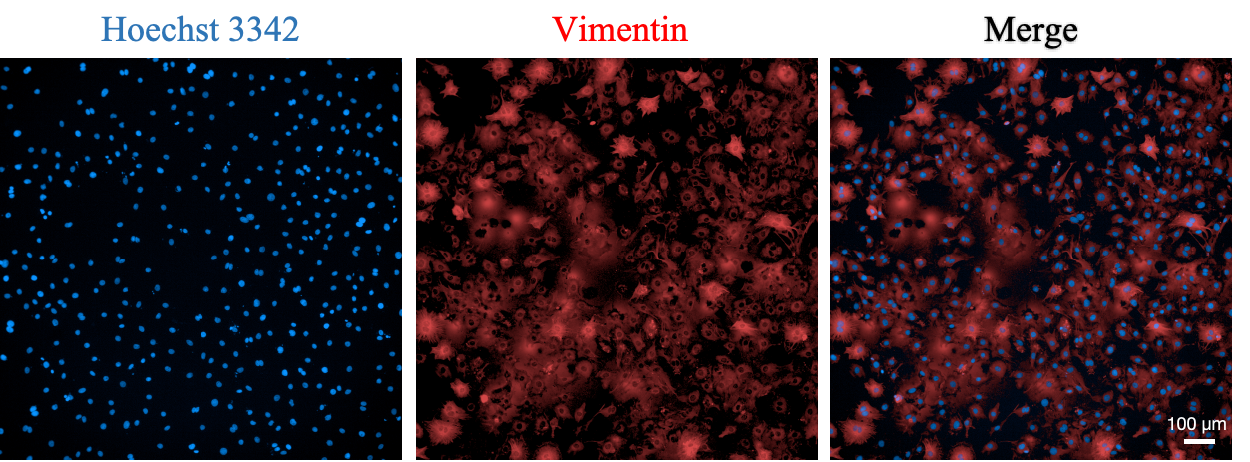


Fig. S1 Operetta image of cellular immunofluorescence
